# Evaluation of Rapid Multiplex Reverse Transcription-Quantitative Polymerase Chain Reaction Assays for SARS-CoV-2 Detection in Individual and Pooled Samples

**DOI:** 10.3390/life13081717

**Published:** 2023-08-10

**Authors:** Young-Hyun Baek, Min-Young Park, Ho-Jae Lim, Dong-Jae Youm, Youngshin You, Seojin Ahn, Jung-Eun Park, Min-Jin Kim, Sun-Hwa Lee, Yong-Hak Sohn, Yong-Jin Yang

**Affiliations:** 1 Department of Molecular Diagnostics, Seegene Medical Foundation, Seoul 04805, Republic of Korea; baek0h@mf.seegene.com (Y.-H.B.); pyli186@mf.seegene.com (M.-Y.P.); 52rotc.hjl@mf.seegene.com (H.-J.L.); ehdwo01@mf.seegene.com (D.-J.Y.); ysyou@mf.seegene.com (Y.Y.); seojin.ahn@mf.seegene.com (S.A.); lithium2864@mf.seegene.com (M.-J.K.); medsohn@mf.seegene.com (Y.-H.S.); 2 Department of Integrative Biological Sciences & BK21 FOUR Educational Research Group for Age-Associated Disorder Control Technology, Chosun University, Gwangju 61452, Republic of Korea; jepark@chosun.ac.kr; 3 Department of Laboratory Medicine, Seegene Medical Foundation, Seoul 04805, Republic of Korea; lshkim@mf.seegene.com

**Keywords:** COVID-19, rapid RT-qPCR, SARS-CoV-2, infection management

## Abstract

Although coronavirus disease 2019 (COVID-19) is no longer a Public Health Emergency of International Concern (PHEIC), severe acute respiratory syndrome coronavirus 2 (SARS-CoV-2) infection has had a vast impact to date. Hence, continuous management is required, given the uncertainty caused by the potential evolution of SARS-CoV-2. Reverse transcription-quantitative PCR (RT-qPCR) diagnosis has been fundamental in overcoming this issue. In this study, the performances of two rapid RT-qPCR assays (Real-Q Direct SARS-CoV-2 Detection Kit and Allplex™ SARS-CoV-2 fast PCR Assay) with short PCR times were comparatively evaluated using a STANDARD M nCoV Real-Time Detection Kit (STANDARD M, conventional RT-qPCR assay). All kits showed a limit of detection values (10^2^–10^3^ copies/reaction). The evaluation showed that the two rapid assay tests had ≥97.89% sensitivity and ≥99.51% specificity (κ = 0.98) for individual samples and ≥97.32% sensitivity and ≥97.67% specificity for pooled samples compared to STANDARD M. These results indicate that the two rapid RT-qPCR kits, which showed significant time reduction in performance, are as effective as a conventional RT-qPCR assay. They are likely to increase not only the number of tests that can be performed but also the efficiency of sustainable management of COVID-19 in the long term.

## 1. Introduction

Coronavirus disease 2019 (COVID-19), caused by severe acute respiratory syndrome coronavirus 2 (SARS-CoV-2), was first reported in December 2019 [1]. Owing to its highly infectious nature, SARS-CoV-2 has spread rapidly worldwide [2,3]. According to the COVID-19 weekly epidemiological data from the World Health Organization (WHO), there were approximately 765 million confirmed cases and 6.9 million associated deaths as of 11 May 2023 [4]. During the COVID-19 pandemic, SARS-CoV-2 evolved through genetic mutations and viral recombination, thereby becoming more transmissible, infectious, and lethal [5]. As of 5 May 2023, COVID-19 is not considered a Public Health Emergency of International Concern (PHEIC), owing to a decline in the mortality rate, decrease in hospitalization rate, and high level of population immunity against SARS-CoV-2 [6,7]. Nonetheless, SARS-CoV-2 remains a global concern because of its potential to evolve; hence, long-term management of COVID-19 is necessary [8,9]. In this milieu, various studies are still being conducted for the accurate and rapid diagnosis of SARS-CoV-2 infection [10,11].

The guidelines for accurate and rapid diagnostic testing of SARS-CoV-2 infection have been proposed by the Centers for Disease Control and Protection (CDC). Various viral diagnostic tests are being used to detect SARS-CoV-2 infection and provide appropriate medical care; these include (1) antigen tests, (2) nucleic acid amplification tests (NAATs), and (3) other additional tests (e.g., breath tests) [12]. Currently, the most commonly used test methods are antigen tests and NAATs, owing to their numerous advantages and few disadvantages [13].

Antigen tests can be used for the rapid prevention of community-acquired infections by enabling quick identification of SARS-CoV-2 infection (approximately 15 min of test time) [14]. However, the sensitivity of antigen tests is lower than that of NAATs, especially in asymptomatic patients [15,16]. In contrast to the antigen tests, the reverse transcription-quantitative polymerase chain reaction (RT-qPCR), an NAAT, has the highest sensitivity and is the most widely used [10]. However, the time required is longer than that for other tests, owing to the time required for nucleic acid extraction and amplification [17]. Therefore, studies have aimed to shorten the assay time by focusing on pooled testing, nucleic acid extraction, and PCR amplification [18,19,20].

First, pooled testing, which is based on the high sensitivity of RT-qPCR, enables one to perform multiple tests simultaneously, thereby saving time [21,22]. The process involves combining the same type of specimens from different individuals and conducting one RT-qPCR assay of the pooled specimens to detect SARS-CoV-2 [23]. Increasing the pool size of samples can reduce the duration of testing, although a low viral load can lead to false negative results [24]. Second, nucleic acid extraction is a prerequisite for the performance of RT-qPCR assays, and the extraction method used affects the results [25]. The performance of manual and automated extraction methods is similar, but automated extraction methods can be used to process many samples quickly [26]. Furthermore, a previous study evaluated automatic nucleic acid extraction equipment with similar performances and found that the extraction time differed among the equipment [27]. Finally, the average PCR amplification time with the conventional RT-qPCR assay kits is 2 h; however, rapid RT-qPCR assay kits that take less than 1 h for assay have been developed by shortening the PCR amplification time [11]. Recently, a rapid RT-qPCR-based kit has been commercialized for the rapid detection of SARS-CoV-2. However, the performance of these rapid assay kits has not yet been compared with that of a conventional RT-qPCR kit, especially when testing pooled samples.

In this study, we evaluated two RT-qPCR kits designed for the rapid detection of SARS-CoV-2, namely, Real-Q Direct SARS-CoV-2 Detection Kit (Real-Q Direct) and Allplex™ SARS-CoV-2 fast PCR Assay (Allplex™ fast). The assay time of these two rapid kits is less than 1 h, and both allow pooled testing. The performance of these rapid assay kits was compared with that of the STANDARD M nCoV Real-Time Detection Kit (STANDARD M), which is widely used for SARS-CoV-2 detection. The diagnostic performance of STANDARD M has already been verified [28,29]. The comparative evaluation allowed us to assess the analytical and clinical effectiveness of the two rapid kits when testing both individual and pooled samples. 

## 2. Materials and Methods

### 2.1. Clinical Specimen Collection and Preparation

Residual nasopharyngeal swab (NPS) specimens (*n* = 1133) were obtained during routine diagnostic testing from November 2021 to August 2022 and evaluated using the SARS-CoV-2 molecular assay in Seegene Medical Foundation (Seoul, South Korea). This study was reviewed and approved by the Seegene Medical Foundation Institutional Review Board (approval number: SMF-IRB-2021-022) with the condition that samples tested positive for SARS-CoV-2 be destroyed after the study. The specimens were used to evaluate the performance of all three SARS-CoV-2 RT-qPCR assay kits. All specimens were anonymized, and all tests were performed in Class-II Biosafety cabinets in accordance with safety regulations [30].

### 2.2. Nucleic Acid Extraction

Total nucleic acids were extracted from all NPS specimens simultaneously (individual and pooled samples) using the MagNA Pure 96 instrument (Roche, Inc., Basel, Switzerland). The MagNA Pure 96 DNA and Viral NA Small Volume Kit and the Pathogen Universal 200 protocol were used. A total of 200 μL of each sample was transferred to the process cartridge for extraction, and the elution volume was set to 100 μL. The extracted nucleic acids were stored at –20 °C to maintain stability.

### 2.3. Selection of NPS Specimens Pooling and Generation of Pooled Samples

NPS specimens for pooling were selected based on the results of individual SARS-CoV-2 tests performed using the three SARS-CoV-2 RT-qPCR assay kits. Overall, 50% of the samples (261/522) whose positive results were confirmed in individual test runs were selected randomly. Pooled samples were generated by mixing one confirmed SARS-CoV-2-positive NPS specimen (100 μL) with four confirmed SARS-CoV-2-negative NPS specimens (100 μL each) in a tube (final pooled sample volume, 500 μL) [18]. 

### 2.4. Multiplex RT-qPCR Assay

All assays were performed using the CFX96 instrument (Bio-Rad Laboratories, Inc., Irvine, CA, USA). The assays were conducted according to the manufacturer’s protocol (Table 1). The STANDARD M kit (SD Biosensor, Suwon, South Korea) targets two SARS-CoV-2 genes, namely, RNA-dependent RNA polymerase (*RdRP*) and envelope (*E*) genes. The RT-qPCR running time for all samples was 1 h 26 min. The total volume of the reaction mixture was 30.5 μL (10 μL of extracted RNA and 20.5 μL of master mix).

The Real-Q Direct kit (BioSewoom, Seoul, South Korea) targets two SARS-CoV-2 genes, namely, *RdRP* and *E*. The RT-qPCR running times for individual and pooled samples were 49 min and 1 h 12 min, respectively. The total volume of the reaction mixture was 25 μL (5 μL of extracted RNA and 20 μL of master mix).

The Allplex™ fast kit (Seegene, Seoul, South Korea) targets three SARS-CoV-2 genes, namely, *E*, *RdRP*, and nucleocapsid (*N*) gene. The RT-qPCR running time was 53 min based on the equipment used for all samples. The total volume of the reaction mixture was 20 μL (5 μL of extracted RNA and 15 μL of master mix).

### 2.5. Interpretation of RT-qPCR Results

Graphs of target gene fluorescence were confirmed to approximate normal amplification curves for all samples. The results of individual samples were analyzed with baseline thresholds set according to each manufacturer’s protocol. Even if one target gene was not detected, the result was considered negative for assays performed with all kits [31]. Of these, the *E* of the STANDARD M kit was represented by the individual cut-off values from the rapid RT-qPCR assay kits in this study. For pooled samples, the detection of at least one gene was considered a positive result [32].

### 2.6. Analytical Performance and Limit of Detection

To compare the analytical sensitivity and limit of detection (LoD) of the different SARS-CoV-2 assay kits, a standard curve was constructed using template RNA (Reference No: NCCP 43382, National Culture Collection for Pathogens, South Korea). Virus-derived RNA isolated from respiratory specimens of individuals infected with SARS-CoV-2 B.1.351 via cell culture was used for this analysis. RNA concentration was determined using the Nanodrop™ One spectrophotometer (Thermo Fisher Scientific, Waltham, MA, USA) and copy number was determined using the following formula:

(1) $$\mathrm{RNA}\;\mathrm{genome}\;\mathrm{copy}\;\mathrm{number}=\left[\frac{\mathrm{RNA}\;\mathrm{concentration}\left(g/\mathrm{mL}\right)}{\mathrm{nt}\;\mathrm{transcript}\;\mathrm{length}\times 340}\right]\times 6.02\times 10^{23}$$

The LoD was defined as the lowest number of RNA genome copies in a reaction detected through the assays with a 95% probability. Considering that the total RNA volume was different for the assays performed with the three kits, the standard curve was obtained in the copies/reaction (rxn) format. The LoD was determined based on 24 replicates over 8 concentrations (ranging from 10^2^ to 10^9^ copies/rxn), prepared via 10-fold dilutions.

### 2.7. Statistical Analysis

SPSS Statistics version 27 (IBM, NY, USA) was used for all analyses and for displaying all graphs. Amplification efficiency was calculated from the linear regression slope using the following formula: E value (%) = 100 × (−1 + 10^−1/slope^) [33]. R^2^ of the standard curves was estimated from the analytical sensitivity results. Probit regression analysis was used to obtain 95% LoD values [34]. The clinical performance of the rapid RT-qPCR assay kits was compared with that of the STANDARD M kit. ROC curves were used to estimate the diagnostic performance of rapid kits using area under curve (AUC) scores. The cut-off values were determined by the ROC curve and the Youden index method. Bland–Altman plot analysis was used to check the differences in the Ct values among the assay kits [35]. The scatter plots were evaluated using linear regression between individual and pooled samples from average Ct values. For each assay kit, the mean Ct values (± standard deviation) that changed when one SARS-CoV-2-positive specimen was pooled with four negative specimens were compared.

## 3. Results

### 3.1. Evaluation of the Analytical Performance and LoD of the RT-qPCR Kits 

**Figure 1:**
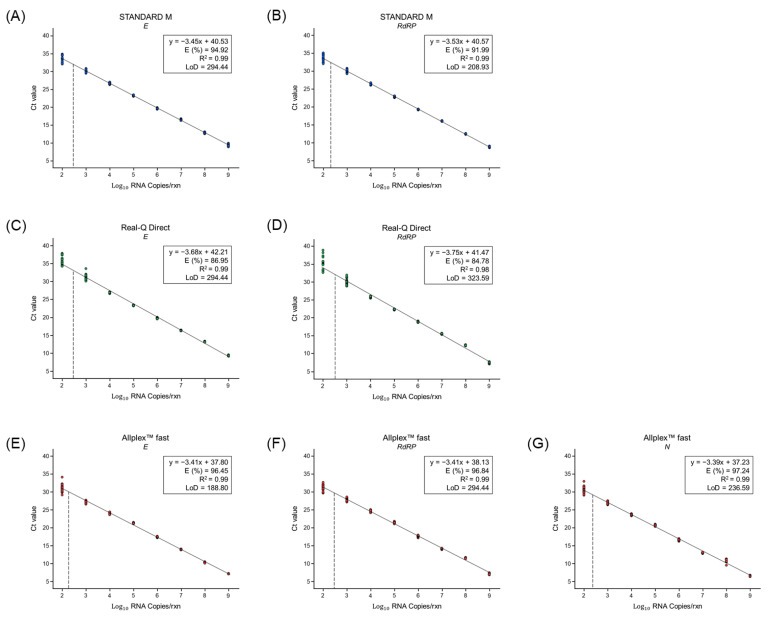
Correlation between the Ct values and SARS-CoV-2 B.1.351 RNA at eight concentrations, as analyzed using linear regression based on LoD experiments. Graphs prepared using the results of the STANDARD M kit for (**A**) *E* and (**B**) *RdRP*; the Real-Q Direct kit for (**C**) *E* and (**D**) *RdRP*; and the Allplex™ fast kit for (**E**) *E*, (**F**) *RdRP*, and (**G**) *N*. Percentage efficiency was calculated from the slope using the formula E (%) = 100 × (−1 + 10^−1/slope^). Circles represent the results of each test, and 24 replicates were used per concentration. The dotted line represents the LoD value. *E*, envelope gene; *RdRP*, RNA-dependent RNA polymerase gene; *N*, nucleocapsid gene; Ct, Cycle threshold; and LoD, limit of detection.

The analytical performance of the STANDARD M, Real-Q Direct, and Allplex™ fast kits was determined using SARS-CoV-2 B.1.351 RNA (Figure 1). The R^2^ values for all RT-qPCR assay kits were ≥0.98, demonstrating consistency and reliability. The amplification efficiency of the three RT-qPCR assay kits for SARS-CoV-2 B.1.351 was between 80% and 120%, which is consistent with the criterion for efficient multiplex RT-qPCR. 

Regarding the LoD, all target genes (*E* and *RdRP* for the STANDARD M kit; *E* and *RdRP* for the Real-Q Direct kit; *E*, *RdRP*, and *N* for the Allplex™ fast kit) were detected at a rate of 100% (from 10^3^ to 10^9^ copies/rxn) by all three assay kits. Thus, the LoD values were as follows: 208.93–294.44 copies/rxn for the STANDARD M kit, 294.44–323.59 copies/rxn for the Real-Q Direct kit, and 188.80–294.44 copies/rxn for the Allplex™ fast kit. These results indicate that the analytical performances of the two rapid kits were similar to that of the STANDARD M kit.

### 3.2. Comparison of the Conventional and Rapid RT-qPCR Kits Using Individual Clinical Samples

A total of 1133 clinical samples were used to compare the diagnostic performance of the rapid RT-qPCR assay kits. The AUCs of the Ct values, which were used to discriminate between PCR-positive and PCR-negative results, ranged from 0.96 to 0.97. The highest optimal viral load cut-off was 30.27 for the Real-Q Direct and 30.81 for the Allplex™ fast kits (Table 2). The sensitivities for Ct values below the cut-off ranged from 85.71% to 91.00% (Supplementary Table S1). Overall, the sensitivity, specificity, PPV, and NPV of the two rapid RT-qPCR kits (Real-Q Direct kit and Allplex™ fast kit) showed similar values to those of the STANDARD M kit (Table 3). The positive coincidence rate of the STANDARD M and the two rapid RT-qPCR kits was 97.13% (507/522). There was a strong correlation (of κ = 0.98) between the clinical performance of the STANDARD M and the two rapid RT-qPCR kits.

### 3.3. Differences in the Ct Values for Clinical Samples between the Conventional and Rapid RT-qPCR Kits

**Figure 2:**
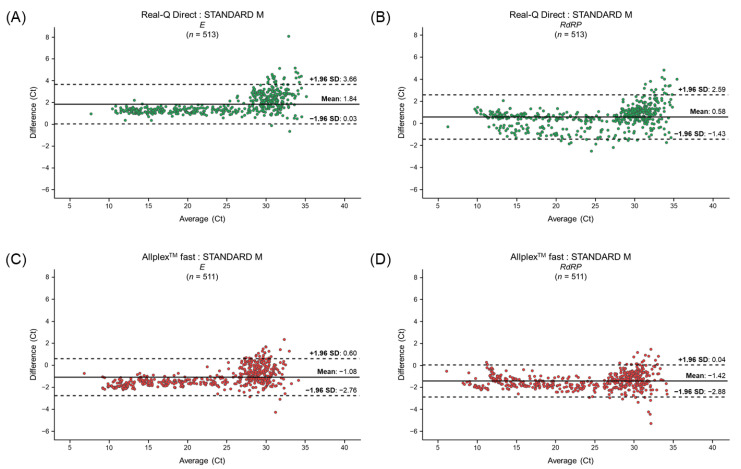
Bland–Altman plot analysis of the differences in Ct values for the same target genes, as tested with the STANDARD M and rapid RT-qPCR kits (Real-Q Direct kit for (**A**) *E* and (**B**) *RdRP*; Allplex™ fast kit for (**C**) *E* and (**D**) *RdRP*). The *x*-axis represents the average Ct value for the STANDARD M and rapid RT-qPCR kits. The *y*-axis shows the difference in the Ct value between the STANDARD M and two rapid RT-qPCR kits. *E*, envelope gene; *RdRP*, RNA-dependent RNA polymerase gene; Ct, cycle threshold; and SD, standard deviation.

We examined the correlations among Ct values obtained when testing a panel of samples (Figure 2). Among the samples confirmed positive with both rapid assay kits and the STANDARD M, we analyzed the Ct values of 513 samples that tested positive with the Real-Q Direct kit and 511 that tested positive with the Allplex™ fast kit. The Ct value for the Real-Q Direct kit assay was higher than that for the STANDARD M kit assay, whereas the Ct value for the Allplex™ fast kit assay was lower than that for the STANDARD M kit assay. The 95% limit of agreement between the Real-Q Direct and STANDARD M kits was 94.93% (487/513 samples) for *E* (Ct difference range: 0.03–3.66) and 94.35% (484/513 samples) for *RdRP* (Ct difference range: −1.43–2.59). The 95% limit of agreement between the Allplex™ fast and STANDARD M kits was 95.11% (486/511 samples) for *E* (Ct difference range: −2.76–0.60) and 94.32% (482/511 samples) for *RdRP* (Ct difference range: −2.88–0.04). Thus, a comparison of the same target genes using the rapid kits and the STANDARD M kit revealed that the value was close to 95%, thereby confirming the similar performance of the two rapid kits and the STANDARD M kit.

### 3.4. Comparative Evaluation of Pooled Samples Using the Two Rapid Assay Kits

**Figure 3:**
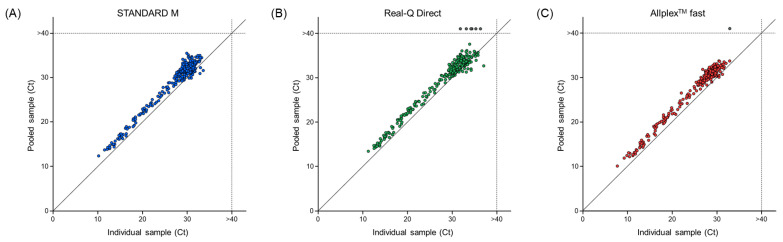
Comparison of Ct values between individual and pooled samples, as tested with the (**A**) STANDARD M, (**B**) Real-Q Direct, and (**C**) Allplex™ fast kits. Samples that were negative are marked with gray dots beyond the dotted line. The *x*-axis represents the average Ct value of individual samples, and the *y*-axis represents the average Ct value of pooled samples. Ct, cycle threshold.

To evaluate the performance of the two rapid RT-qPCR assay kits using pooled samples, 261 samples that tested positive in individual test runs with all assay kits were randomly selected. Overall, 97.70% (255/261) of the samples tested positive with the Real-Q Direct kit and 99.62% (260/261) tested positive with the Allplex™ fast kit. When individual positive samples were pooled, the average Ct value for the STANDARD M, Real-Q Direct, and Allplex™ fast kits increased by 1.96 ± 1.11, 1.45 ± 1.14, and 2.20 ± 0.84, respectively (Figure 3). 

### 3.5. Clinical Samples Showing Discordant Results for Pooled Samples

After comparing the results of pooled samples evaluated using the rapid assay kits with those evaluated using the STANDARD M kit, six samples showed discordant results. Specifically, all six samples were positive when assayed using the STANDARD M kit, but negative when assayed using the Real-Q Direct kit. In addition, one sample was negative when assayed using either of the two rapid RT-qPCR assay kits (sample F; Table 4). There was a difference in the number of discordant results recorded with each rapid RT-qPCR assay kit, but it was insignificant (*p* > 0.05).

## 4. Discussion

The highly infectious SARS-CoV-2, the causative pathogen of COVID-19, remains a serious threat to healthcare systems worldwide [36]. Recently, the Omicron sub-variant has emerged as the predominant strain [7]. Mutations increased the infectivity of the virus, causing major disruptions in global healthcare systems and a shortage of reagents and materials for testing [26]. Fortunately, high population-level immunity and vaccination have contributed to a substantial decrease in the number of confirmed cases and deaths worldwide since the start of the pandemic [7]. However, the possibility of the evolution of SARS-CoV-2 and global risk remains high [8]. Therefore, the development of a rapid and effective diagnostic method for SARS-CoV-2 infection is crucial for the sustainable management of the virus [10].

Currently, NAATs and antigen diagnostic tests are used to detect SARS-CoV-2 infection and provide appropriate medical services [13]. RT-qPCR is a gold standard method for detecting SARS-CoV-2 infection [37]. Loop-mediated isothermal amplification (LAMP) PCR, a type of NAAT, takes less time than RT-qPCR assays, but results in a high rate of false negatives [38]. Rapid antigen tests have the advantage of the shortest assay time compared to other tests. However, numerous studies have reported the limitations of rapid antigen testing owing to its lower clinical performance in comparison with that of RT-qPCR [39,40].

The conventional RT-qPCR method requires the longest assay time. If the time required for the RT-qPCR test can be reduced, continuous and effective management of patients with COVID-19 can be realized. Rapid RT-qPCR assay can play an important role in achieving this goal. Therefore, the aim of the present study was to evaluate the performance of two rapid RT-qPCR assay kits using individual and pooled samples with various Ct values.

Here, we evaluated the analytical performance through serial dilutions of SARS-CoV-2 B.1.351 RNA. For all target genes, the RT-qPCR amplification efficiency was within the reference range of 80–120%, and the LoD range was similar (Figure 1). These results confirmed that the analytical performance of the Real-Q Direct and Allplex™ fast kits was similar to that of the STANDARD M kit.

Based on the similar analytical performance, the clinical performance of the two rapid RT-qPCR kits was evaluated using 1133 individual clinical samples (Table 2 and Table 3). For the Real-Q Direct kit, the sensitivity was 98.28% and the specificity was 99.51%. For the Allplex™ fast kit, the sensitivity was 97.89% and the specificity was 99.84%. The clinical performance of each rapid RT-qPCR kit and STANDARD M kit showed a high association (κ = 0.98). In addition, the distribution of differences in the results was analyzed based on the same target genes (*E* and *RdRP*), focusing on the average Ct values of the STANDARD M kit and each rapid RT-qPCR kit (Figure 2). It was confirmed that approximately 95% of the similarity values originated from the Real-Q Direct and Allplex™ fast kits around the average.

Compared to the STANDARD M kit, the Real-Q Direct and Allplex™ fast kits detected 97.70% (255/261) and 99.62% (260/261) of positive cases in the pooled sample (Figure 3). When the samples are pooled, the Ct value should theoretically increase. Overall, it was found that the average Ct value of the pooled samples increased by an average of 1.45–2.20 compared with that of the individual samples when assayed with all RT-qPCR assay kits. Conversely, we also found cases where the Ct values of the pooled samples were lower than those in the individual samples. They occurred at a Ct value above 30, probably because the cut-off value was approached or the suppression effect was reduced by dilution. However, in pooled sample testing, the high clinical performance of the two rapid RT-qPCR assay kits compared with that of the STANDARD M kit was confirmed. Therefore, the two rapid RT-qPCR kits and the conventional RT-qPCR kit have similar analytical and clinical performances for both individual and pooled samples.

In pooled sample testing, six samples showed discordant results between the STANDARD M kit and the Real-Q Direct kit, and one between the STANDARD M kit and the Allplex™ fast kit (Table 4). This difference can be explained as follows. In pooled sample testing, individual tests were performed even if only one target gene was detected in the pool. Thus, the Allplex™ fast kit would have been more advantageous than the Real-Q Direct kit in the pooled sample testing because it detects an additional target gene. In addition, pooled sample testing could only be completed in less than 1 h based on PCR amplification time with the Allplex™ fast kit. Owing to the shortest PCR running time and the smallest number of discordant samples, we suggest that the Allplex™ fast kit is the most suitable for conducting large-scale screening tests.

Based on our results, the analytical and clinical performances of the Real-Q Direct and the Allplex™ fast kits for both individual and pooled samples were found to be similar to those of the STANDARD M kit. These rapid RT-qPCR kits can help healthcare professionals make quick and informed decisions by reducing the diagnosis time of COVID-19. Also, they can help reduce the virus exposure time of patients and medical staff, especially in emergency departments. From an economic point of view, these rapid kits can reduce costs associated with COVID-19 patient isolation and waiting times. In addition, it can increase the number of tests performed without additional equipment or space. Furthermore, it makes it possible to save time, workforce, and resources related to increasing diagnosis capacity. If there are COVID-19 outbreaks in the future, this diagnostic method will play a major role in global medical surveillance systems. Through this diagnostic method, COVID-19 can be managed sustainably and effectively in the long term. 

## 5. Conclusions

Rapid and accurate diagnosis is essential for the efficient management of COVID-19. RT-qPCR is the most sensitive method for COVID-19 diagnosis, but it takes longer time than other methods. We evaluated two rapid RT-qPCR kits, which shortened the PCR amplification time to less than 1 h, and confirmed that their performance was similar to that of the conventional RT-qPCR kit when testing individual and pooled samples. The global burden of COVID-19 is decreasing, but we should prepare for sustainable management of the disease post-pandemic. We expect that the two rapid RT-qPCR kits with reduced processing time and high accuracy will be useful diagnostic tools for the management of COVID-19.

## Figures and Tables

**Table 1:** Specifications and PCR conditions for the three commercial kits used for SARS-CoV-2 detection.

Kit	STANDARD M nCoV Real-Time Detection Kit	Real-Q Direct SARS-CoV-2 Detection Kit	Allplex™ SARS-CoV-2 Fast PCR Assay
Target genes	*E*, *RdRP*	*E*, *RdRP*	*E*, *RdRP*, *N*
Template volume	10 μL	5 μL	5 μL
PCR running time (individual)	1 h 26 min	49 min	53 min
PCR running time (pooled)	1 h 26 min	1 h 12 min	53 min
SARS-CoV-2 Variants (lineage)	16 strains	N/A	20 strains
Interpretation (individual)	Positive—All target genes detected Negative—Even if one target gene is not detected		
Interpretation (pooled)	Positive—Even if one target gene is detected Negative—No target genes detected		

The STANDARD M kit was used as a reference for evaluating the Real-Q Direct and Allplex™ fast kits. Abbreviations: *E*, envelope gene; *RdRP*, RNA-dependent RNA polymerase gene; and *N*, nucleocapsid gene.

**Table 2:** Performance of two RT-qPCR kits in detecting SARS-CoV-2 (based on STANDARD M Ct values).

Target	Assay	AUC	95% CI	*p*-Value	Cut-Off
*E*	Real-Q Direct	0.96	0.91–1	<0.001	30.27
	Allplex™ fast	0.97	0.94–0.99	<0.001	30.81
*RdRP*	Real-Q Direct	0.97	0.93–1	<0.001	32.71
	Allplex™ fast	0.97	0.95–0.98	<0.001	32.44

Data indicate the receiver operating characteristic curve for target gene from rapid RT-qPCR kits. Abbreviation: AUC, area under curve; CI, confidence interval.

**Table 3:** Evaluation of the clinical sensitivity and specificity for individual samples.

Assay	STANDARD M				Sensitivity % (95% CI)	Specificity % (95% CI)	PPV % (95% CI)	NPV % (95% CI)	κ Value
	TP	FP	FN	TN					
Real-Q Direct	513	3	9	608	98.28 (96.75–99.21)	99.51 (98.57–99.90)	99.42 (98.31–99.88)	98.54 (97.25–99.33)	0.98
Allplex™ fast	511	1	11	610	97.89 (96.26–98.94)	99.84 (99.09–100)	99.80 (98.92–100)	98.23 (96.85–99.11)	0.98

Abbreviations: TP, true positive; FP, false positive; FN, false negative; TN, true negative; PPV, positive predictive value; NPV, negative predictive value; and CI, confidence interval.

**Table 4:** Details of discordant results and Ct values from pooled clinical samples.

Sample	STANDARD M			Real-Q Direct			Allplex™ Fast			
	*E*	*RdRP*	Result	*E*	*RdRP*	Result	*E*	*RdRP*	*N*	Result
A	34.36	33.35	Detected	-	-	Not detected	33.60	-	32.32	Detected
B	34.00	35.01	Detected	-	-	Not detected	34.16	31.76	-	Detected
C	34.55	34.38	Detected	-	-	Not detected	33.51	32.62	-	Detected
D	34.62	34.53	Detected	-	-	Not detected	-	-	32.21	Detected
E	34.02	34.2	Detected	-	-	Not detected	-	-	33.17	Detected
F	-	34.64	Detected	-	-	Not detected	-	-	-	Not detected

Abbreviations: RdRP, RNA-dependent RNA polymerase gene; E, envelope gene; and N, nucleocapsid gene.

## Data Availability

All data are available within the article.

## Supplementary Materials

The following are available online at https://www.mdpi.com/article/10.3390/life13081717/s1. Supplementary Table S1: Evaluation of the clinical performance of cut-off value in individual samples.
