# Supplementary material for: Evaluation of Rapid Multiplex Reverse Transcription-Quantitative Polymerase Chain Reaction Assays for SARS-CoV-2 Detection in Individual and Pooled Samples
Source: Life (Basel). 2023 Aug 10;13(8):1717. doi: 10.3390/life13081717 (PMC10455980; doi:10.3390/life13081717)
Supplement: Supplementary file 1 [file life-13-01717-s001.zip › life-2520464-supplementary.pdf]

life

Supplementary Materials

Supplementary Table S1. Evaluation of the clinical performance of cut-off value in individual samples.

Assay	Cut-Off	STANDARD M				Sensitivity % (95% CI)	PPV % (95% CI)
		TP	FP	FN	TN		
Real-Q Direct	Ct<30.27	422	1	-	-	100 (99.13–100)	99.8 (98.69–99.99)
	30.27≤Ct	91	2	9	608	91.00 (83.60–95.80)	97.85 (92.45–99.74)
Allplex™ fast	Ct<30.81	445	1	-	-	100 (99.17–100)	99.8 (98.76–99.99)
	30.81≤Ct	66	-	11	610	85.71 (75.87–92.65)	100 (94.56–100)
